# Intraosseous Lipoma: A Report of a Case in the Mandibular Symphysis Region

**DOI:** 10.7759/cureus.23658

**Published:** 2022-03-30

**Authors:** Carlo Maksoud, Georges Aoun

**Affiliations:** 1 Oral Medicine and Maxillofacial Radiology, Lebanese University, Beirut, LBN

**Keywords:** viable fat cells, necrotic fat, mandibular symphysis, intraosseous lipoma, bone lesion

## Abstract

Intraosseous lipomas are benign lesions of the bone. In the jaws, they are very rare and in most cases incidentally discovered on panoramic radiographs taken in dental practice. They are usually asymptomatic and appear radiologically as a radiolucent image sometimes including some radio-opacities. Histologically, they consist of mature adipose tissue associated with variable degrees of necrotic fat and calcification. In this report, we describe a case of intraosseous lipoma in the mandibular symphysis region of a 37-year-old female as well as the treatment adopted.

## Introduction

Lipomas are common, benign tumors of adipose tissue that may involve any part of the body, especially the trunk and proximal extremities. They generally present as slow-growing soft masses. Usually, lipomas are asymptomatic when they are subcutaneous, and may cause pressure-related symptoms when they are deep [1]. Although adult bone marrow is largely fatty, intraosseous lipomas (IOLs) are extremely rare [2,3]. They are mostly detected in the long bones (femur, fibula, tibia, radius, and humerus) and calcaneus [1,2]. Very few cases in the literature were reported in the jaws.

Milgram has proposed a histological classification of IOLs depending on the degree of the lesion evolution: Stage 1, a tumor with viable fat cells; stage 2, a tumor composed of both viable fat cells and fat necrosis, and calcification; stage 3, a tumor with necrotic fat, calcification of necrotic fat, variable degrees of cyst development, and reactive new bone formation [4].

Radiologically, their appearance can vary according to the stage of the lesion [5,6]. Typically, they present as osteolytic bone lesions with well-defined margins. Central calcification may be noticed, thus creating the cockade sign [7]. Computed tomography (CT) and magnetic resonance imaging (MRI) techniques can be helpful in detecting fat (viable/necrotic) and calcification within the lesion, therefore allowing a more precise diagnosis [8]. MRI features of IOLs include, similar to adipose tissue, high signal intensity on both T1 and T2-weighted images [3]. In this report, we describe a rare case of a 37-year-old female with mandibular IOL.

## Case presentation

**Figure 1:**
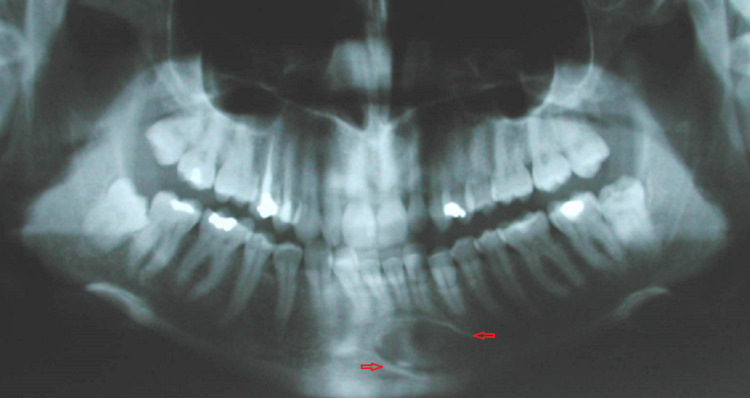
Panoramic radiograph showing radiolucency located in the anterior region of the mandible below the teeth apices

A 37-year-old female was referred by her dentist to our oral and maxillofacial surgery center for evaluation of a radiolucency incidentally detected on a panoramic radiograph. The lesion was located in the anterior region of the mandible below the teeth apices (Figure 1).

Medical and physical examinations revealed a healthy woman with no extra-oral abnormal findings. Intra-orally, no signs of inflammation were found and the mandibular anterior teeth were positive to vital tests.

**Figure 2:**
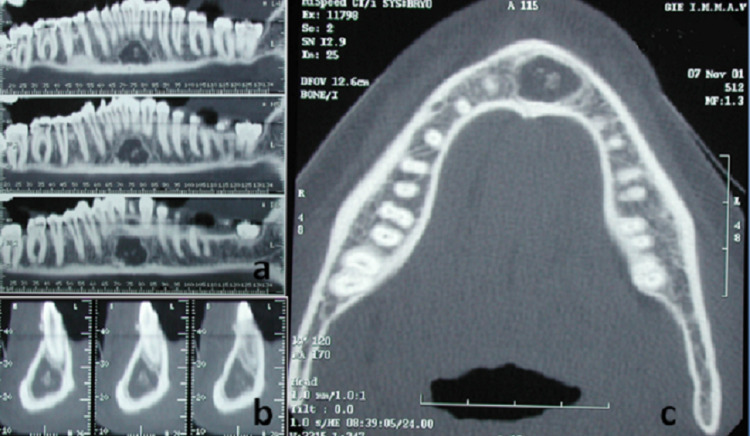
CT scan of panoramic reconstruction (a), cross-sectional (b), and axial (c) cuts showing a well-defined unilocular hypodense image centered by small round radiopacities located in the mandibular symphysis region

A CT scan was requested for better assessment of the lesion. Panoramic reconstruction, cross-sectional and axial views revealed a well-defined unilocular hypodense image centered by small round radiopacities located in the mandibular symphysis region. No buccal and lingual plates’ expansion or root resorptions or displacement were noticed (Figures 2a-2c).

Under loco-regional anesthesia, a vestibular mucoperiosteal flap was detached from the underlying bone for better visibility and the cortical plate was drilled for access window through which a thorough curettage of the lesion was made.

Macroscopically, yellow fat lobules were observed, separated by bony partitions and vascular septa; the dense structures visible on radiographic examination appeared as intralesional bone calcifications.

**Figure 3:**
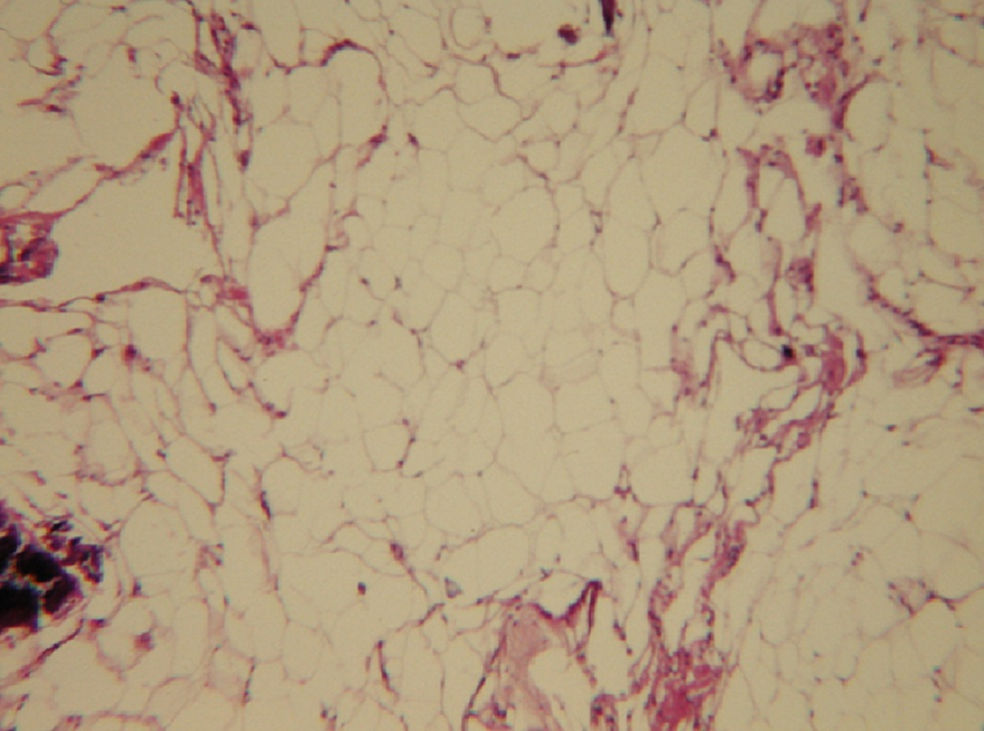
Histological section showing a mature adipose tissue associated with necrotic fat and calcification

Histopathological results of the specimen showed a mature adipose tissue, without cellular atypia, associated with variable degrees of necrotic fat and calcification (Figure 3).

The diagnosis was in favor of an IOL. Annual follow-up is suggested to prevent any recurrence of the lesion.

## Discussion

IOLs are very uncommon benign tumors of the bone that account for about 0.1% of all bone lesions. They are mostly detected in the fourth and fifth decades of life with no gender predilection [1,9,10]. IOLs are rarely found in the jaws [6] and less frequently in the maxilla than in the mandible [9]; not more than 20 cases have been documented in the literature [11] with probably the first one described by Oringer, in 1948, involving the body of the mandible [12].

For many researchers, the etiology of IOLs remains unknown. For others, trauma and nutritional problems may be among the etiological factors. In the mandible, which is supplied principally by the inferior alveolar artery, obliteration of the nutrient arteries may lead to infractions in some areas where fatty cells of bone marrow may collect to form a “lipomatous mass” [13]. For Polte et al. when the vascular component within IOLs is prominent, they are considered to be variants of angioma and so-called angiolipomas. Other histological variants have been reported in the literature including fibrolipomas and spindle cell lipomas [14].

Mandibular IOLs, generally occur in the posterior segment and very rarely in the symphysis region [1,10]. According to Waśkowska et al., in addition to their case, only four other cases located in the anterior section of the mandible were reported since 1948 [15].

The symptoms of IOLs depend on the location and size of the lesion [10]. If symptomatic, patients may suffer from swelling, pain, and paresthesia [1,2]. Differential diagnosis suggests many lesions such as cysts, benign odontogenic tumors, fibrous dysplasia, primary malignant liposarcoma or metastatic tumors, etc. [10]. All these jaw lesions may present comparable radiological images as IOLs and the final diagnosis is confirmed by histopathological examination. Malignant transformation of IOLs should be suspected when rapid bone destruction is noticed [16].

The treatment of choice for IOLs is surgical enucleation. However, extensive lesions may require bone resection followed by grafting [10,13,15]. Recurrence is extremely rare and four cases of malignant transformation were reported in the literature [16].

In our report, we have presented a case of a stage 2 mandibular IOL. A complete surgical enucleation was performed with no recurrence till date.

## Conclusions

Mandibular IOLs are rare bone tumors. There are few reported cases in the literature located in the anterior section of the mandible. Many lesions may constitute a differential diagnosis challenge for IOLs. For that, a thorough assessment of the clinical, radiological, and histological components of these lesions is mandatory to surmount the diagnostic. Furthermore, their early diagnosis and accurate treatment consisting in a complete surgical enucleation/resection followed by long-term follow-up of the patient are essential to prevent any probable recurrence.
